# Factors influencing nutrition care process and nutrition care process terminology implementation among United States dietetics educators and preceptors

**DOI:** 10.3389/fnut.2026.1805075

**Published:** 2026-04-29

**Authors:** Luc R. LaBonte, Constantina Papoutsakis, Sherri Lewis, Casey Colin

**Affiliations:** 1 Department of Nutrition Science, East Carolina University, Greenville, NC, United States; 2 Department of Nutrition and Dietetics, University of North Florida, Jacksonville, FL, United States; 3 Academy of Nutrition and Dietetics, Chicago, IL, United States; 4 James A. Haley Veterans’ Hospital, Tampa, FL, United States

**Keywords:** evidence-based practice, nutrition care process, nutrition education, professional practice, survey

## Abstract

**Objective:**

Identify variables associated with Nutrition Care Process (NCP) and Terminology (NCPT) implementation among United States dietetics educators and preceptors using an online survey.

**Methods:**

The survey was developed using validated tools and distributed via email using a university-hosted Qualtrics platform. Participants were recruited through a mixed sampling strategy including randomized outreach, voluntary participation, and snowball sampling. Data were screened for incomplete responses and outliers prior to inferential analyses. Relationships between relevant variables and implementation intention were analyzed using tests selected based on parametric assumptions.

**Results:**

Of 266 responses, 104 were excluded due to declining consent (2), ineligibility (96), or survey noncompletion (6), yielding a final sample of 162 participants: 89 educators (55%), 52 preceptors (32%), and 21 educator–preceptors (13%). Level of education, *χ*^2^ (2) = 18.26, *p* < 0.001, *V* = 0.337; Academy of Nutrition and Dietetics membership, *χ*^2^ (1) = 10.18, *p* < 0.001, *φ* = 0.251; perceived NCP competency, *χ*^2^ (3) = 30.84, *p* < 0.001, *V* = 0.438; measured proficiency score (PS), *U* = 1998.5, *p* < 0.004, *r* = 0.228; and mean attitude score (MAS), *U* = 2013.5, *p* < 0.005, *r* = 0.221, were significantly associated with NCP/T implementation.

**Conclusion:**

Attitude, perceived competency, assessed proficiency, education level, and Academy membership were significantly associated with NCP/T implementation, demonstrating small-to-medium effects. Although not statistically significant, preceptors showed lower implementation rates and significantly lower MAS and PS than educators. Educators and preceptors working in outpatient settings may be particularly vulnerable to implementation barriers, highlighting the need for targeted support to promote consistent, evidence-informed NCP/T use.

## Introduction

Since its formal adoption by the Academy of Nutrition and Dietetics (Academy) in 2003, the Nutrition Care Process (NCP) has become a cornerstone of standardized nutrition care delivery (1–4). Designed as a structured, systematic approach to care, the four-step model—nutrition assessment and reassessment, diagnosis, intervention, and monitoring and evaluation—supports consistent, high-quality practice across diverse clinical settings. This framework encompasses six interconnected clinical judgments that operate within two core phases: problem identification and problem solving (4). As originally proposed by Splett and Myers (5), the NCP was intended to improve the consistency, accountability, and quality of nutrition care. These intended benefits are closely related to the use of “NCP links”—the explicit connections between key clinical judgments such as evidence, diagnosis, etiology, intervention, goal and outcome (6).

Advancements in informatics, standardized language via the Nutrition Care Process Terminology (NCPT), and data infrastructure have further reinforced the theoretical foundation of the model (4, 7). However, empirical support for the routine use of the NCP and NCPT (NCP/T) has emerged more gradually. Landmark studies by Lewis et al. (8) and Mujlli et al. (9), using the validated Diet-NCP-Audit tool developed by Lövestam et al. (10), have demonstrated clear links between adherence to the NCP and improved patient outcomes. At a Veterans Health Administration facility, Lewis and colleagues (8) found that each one-point increase in audit score was associated with a 38% increase in the odds of resolving a nutrition problem, while documentation of the etiology-intervention connection was associated with a 51-fold increase in the likelihood of problem improvement.

Simultaneously, Mujlli et al. (9) reported significant associations between audit scores and clinical outcomes in pediatric patients with non-alcoholic fatty liver disease and metabolic syndrome in Saudi Arabia. Improved documentation and use of the NCP were correlated with reductions in body mass index and alkaline phosphatase levels over a 6-to-12-month follow-up period. Despite differences in setting and population, both studies underscore the same message: the consistent application of the NCP—and documentation of its chain links—is strongly associated with improved patient care and clinical outcomes.

Additional evidence supporting the necessity of NCP use comes from Colin et al. (11), who analyzed registry data from the Academy of Nutrition and Dietetics Health Informatics Infrastructure (ANDHII). Their findings showed that use of the evidence-diagnosis chain link predicted diagnosis resolution in a national diabetes cohort, reinforcing the broader applicability of NCP-informed documentation and care delivery (12). Together, these findings confirm that structured NCP use is not merely best practice—it is essential for achieving measurable improvements in nutrition-related outcomes.

This growing evidence base highlights a critical shift: use of the NCP/T is no longer optional or aspirational. It is a necessary foundation for professional practice, quality assurance, and patient-centered outcomes. While proficiency in NCP/T application remains important, it is the consistent, systematic use of the model—especially through documented chain links—that drives improved care. As such, promoting the routine and accurate implementation of the NCP/T should be a central priority in nutrition and dietetics practice, education, and policy.

Despite strong evidence supporting its effectiveness, implementation of the NCP/T remains variable across practice settings. Findings from large-scale datasets, including the International Nutrition Care Process Implementation Survey (INIS) and registry-based analyses, suggest that consistent and comprehensive use of the NCP/T is not yet universal, with variability observed across regions, practice environments, and professional roles. International studies further demonstrate that this variability persists across healthcare systems, with differences driven by infrastructure, training, policy, and cultural context (13–15). Emerging evidence indicates that both structural and perceptual determinants (such as training exposure, institutional support, perceived usefulness, and practitioner attitudes) may influence adoption and sustained use (13, 16). Notably, even in settings where formal education includes the NCP, consistent implementation in practice remains inconsistent, highlighting persistent gaps between knowledge and application. However, the relative contribution of these factors remains incompletely understood, particularly among individuals responsible for training future practitioners. This gap underscores the need for further investigation into the determinants of NCP/T implementation within dietetics education contexts.

Although international studies have identified common barriers and variability in NCP adoption, the collective literature has not examined the attitudes, perceptions, implementation intentions, and practices of practitioners involved in formal dietetics education. Desroches and colleagues (17) were the last authors to exclusively research these populations, but the study had a limited number of participants and was focused in one organization in Canada with data collected in 2011. Since that time, the NCP has evolved, NCPT has expanded, terminology has become interoperable, the relationship between NCP/T and evidence-based practice has been boldly established, and validated tools designed to measure various associated constructs have been developed. These updates strongly reinforce the need for a new examination of this population, as this will provide future investigators with quantitative data to support development of targeted educational intervention for improving NCP/T adoption. The purpose of this investigation was to identify variables that influence NCP/T implementation intention among United States dietetics educators and preceptors.

## Methods

### Survey development

The instrument used for this investigation was developed and administered through Qualtrics, version March 2024 (Qualtrics) (18). The survey in its entirety can be found in Supplementary material. Five primary blocks were utilized, including sections for introduction, demographics, NCP/T familiarity and usage, NCP/T proficiency, and attitudes toward the NCP/T. Most of the items in the proposed instrument have been composed of previously validated content from the International Nutrition Care Process and Terminology Implementation Survey, or INIS (19). Five of the eight items (62.5%) in demographics, three of the six items (50%) in NCP familiarity and usage, and all items in the attitude assessment were taken directly from the INIS with minimal or no modification. Items within the familiarity and implementation block were adapted from those offered by INIS; although, minor modifications were implemented to reduce survey duration and participant burden.

The introduction block served to orient interested participants in the study by stating the purpose, desired participants, anticipated time requirement, information management procedures, and other frequently asked questions. The section concluded with electronic consent, communicating the rights of the participant and providing a multiple-choice question with two options that correspond to consenting or not consenting. Participants that selected the option that corresponds with denied consent were directed to the survey conclusion page. Providing consent allowed the participants to move onto the demographics block.

Demographic data was collected to further categorize participants and assess variables that may influence NCP implementation intention. Eight questions (multiple choice with ability to enter unlisted options, multiple selection, drop-down bar selection, and direct text entry) were included. Variables assessed related to participant category, year of practice eligibility, practice areas, state where education was completed, state where practicing, and Academy membership.

Screener questions were present to ensure that the participants were among the target populations. Those not identifying with the intended audience were directed to the end of the survey.

Completion of demographics allowed entry into the NCP/T familiarity and implementation block. This included a variety of question formats such as multiple choice with ability to enter unlisted options, multiple selection, and direct text entry. An initial screener was present, directing those that have not heard of the NCP to the survey conclusion page. The subsequent questions used display logic to target the intended audiences’ NCP/T implementation/intention to implement (and rationale for those that do not), familiarity, perceived competence, and sources of NCP/T education.

Items used for the NCP proficiency block were taken from the recently validated NCP Assessment of Brief Level of Expertise (NCP-ABLE) screen (20). All questions were multiple choice (with one correct answer, three incorrect selections, and one option for “I don’t know or am unsure”) and appeared in a random order. One point was awarded for each correct answer selected. The NCP-ABLE instrument uses a 4-point ordinal scoring system to assess the level of practitioner proficiency in applying the NCP. A proficiency score (PS) of 0 indicates performance suggestive of non**-**proficiency, signaling a need for general competency assessment. A PS of 1 reflects a low level of proficiency, suggesting the practitioner may be beginning to develop knowledge beyond basic competency. A PS of 2 is indicative of mid-level proficiency, with performance more consistent with practitioners functioning at a proficient level. Lastly, a PS of 3 suggests a high level of proficiency, representing performance that may warrant advanced practice or expert-level assessment.

The survey concluded with assessment of NCP/T attitudes. Sixteen statements with randomized order were included, requiring the participant to select their level of agreement with a 5-point Likert scale (from strongly disagree to strongly agree). Each selection was a corresponding point value assignment that increases by one per increase in agreement, with strongly disagree being one and strongly agree being 5. All statements were favorably phrased, meaning that strong agreement resulted in a higher score. An unscored sixth option (“not applicable”) was listed to allow participants the opportunity to opt out of sharing for each statement. The mean attitude score (MAS) was calculated for each participant from the average score of statement responses, indicating that unscored options will not influence the final designation. Completion of this block directed participants to the end of survey page that includes a statement of gratitude and contact information for the principal investigator.

### Survey distribution and collection

The survey was distributed to registered dietitian nutritionists (RDNs) through multiple electronic means containing a summary of the research, the survey deadline, a call-to-action description, an anonymous link to the instrument, and approval statement. Eligibility criteria included maintaining RDN credentials and serving in an education-related role, supervised practice preceptor or university educator, for a United States-based Accreditation Council for Education in Nutrition and Dietetics (ACEND) accredited program, All responses were collected across a four-week span between March and April 2024, with data being stored in a secure drive for future analysis. To ensure adequate sample size and statistical power, follow up analyses using a 95% confidence interval were conducted for each population using the Qualtrics sample calculator (21). According to the joint Academy and Commission on Dietetic Registration (CDR) needs assessment study (22) from 2020, 119,249 RDNs are currently practicing within the United States (with approximately 8% working in a higher education institution as their primary practice setting). Assuming that each of these individuals is either a preceptor or educator while using a 95% CI with a 5% margin of error, the necessary sample size is 370 participants (21).

The first distribution approach was direct email contact. Email addresses were obtained from the CDR randomized registry and the public ACEND program directory. CDR was provided with the message materials and claimed responsibility for disseminating the messages to the randomized registry. Email addresses from the ACEND public directory (23) were collected in a Microsoft Excel document and stored within a secure drive. The document was reviewed for removal of duplicate email addresses or identities. All addresses were transferred into Qualtrics for anonymous dissemination. In addition to the first message containing the previously mentioned content, individuals contacted through this method received two follow-up reminders (one midpoint and another 2 days prior to the collection deadline).

The second distribution mode included posts on external Academy discussion boards. The content communicated in the posts was similar to the email distributions. Follow-up posts were not aligned with etiquette suggested by the forum leadership, resulting in one post on each relevant board on the first day of the collection phase.

### Preliminary data analysis

Preliminary analysis began immediately after the collection phase. Data was exported from Qualtrics into Microsoft Excel for variable recoding. Each variable was reviewed for “Other (please specify)” responses. Responses that did not match offered selections were kept for later review to offer context in the discussion. This coded Microsoft Excel file was saved in a secure drive. This data was imported into IBM SPSS Statistics for Windows version 29.0 for ongoing analysis.

Observations were reviewed for missing responses. Cases with incomplete data were flagged for removal. These observations were removed if less than 5% of total observations were incomplete or missing data is presented in a random pattern (24). Observations with outlier values were flagged for review. Outliers were identified through visualization on graphs and plots as well as descriptive statistics interpretation. Those exceeding three standard deviations were considered outliers from a quantitative perspective (24). Values outside of these ranges were assigned an identical score as the next closest observation within three standard deviations to avoid the loss of statistical impact that would be made through deletion (24). Location data within the United States by state was binned into four regional groups—United States Northeast, United States South, United States Midwest, and United States West (25). State distribution by region can be reviewed in Supplementary material. Base descriptive analyses were completed multiple times throughout the data cleaning process. A final tentative analysis was completed for review.

### Statistical analysis

Descriptive statistics were used to summarize participant characteristics and key study variables. Categorical variables were reported as frequencies and percentages, while continuous and ratio-level variables were summarized using means and standard deviations. The primary outcome variable, intention to implement the NCP/T, was binary (Yes/No) and treated as a nominal variable. To explore associations between this outcome and various independent variables, inferential statistical tests were selected based on the level of measurement of the predictor variables. Tests selected can be seen in Supplementary material.

For nominal independent variables (such as professional role) associations with NCP/T intention were assessed using Chi-square tests of independence. Where expected cell counts were below five, Fisher’s Exact Test was used. Cramér’s *V* was calculated for significant Chi-square tests to estimate effect size. Strength of association for dichotomous variables (such as Academy membership) was measured with Phi (24) Ordinal independent variables (such as Likert-scale measures for perceived competence) emphasized Chi-square tests to assess associations. Ratio-level independent variables (such as individual attitude items) used independent samples t-tests to compare mean values between respondents who did and did not intend to implement the NCP/T. Levene’s Test was used to assess homogeneity of variance. When parametric assumptions were not met, non-parametric alternatives such as the Mann–Whitney *U* test were applied. Effect sizes for inferential analyses were interpreted per Cohen’s guidelines (small ≈ 0.1, medium ≈ 0.3, large ≥ 0.5) (24).

Relevant variables were evaluated for parametric analysis qualification. Distribution of data was evaluated through histogram review, skewness and kurtosis values, and Kolmogorov–Smirnov and Shapiro–Wilk test outputs (24) Independence of the data was assessed through Durbin-Watson testing (24). Equality of variance was determined by Levene’s test and residual plot review when applicable (24). Acceptable level of measure varied by intended analysis and can be seen in Supplementary material. All statistical analyses were performed using SPSS Version 29.0 with two-sided significance levels set at *p* < 0.05.

## Results

### Data preparation

**Figure 1:**
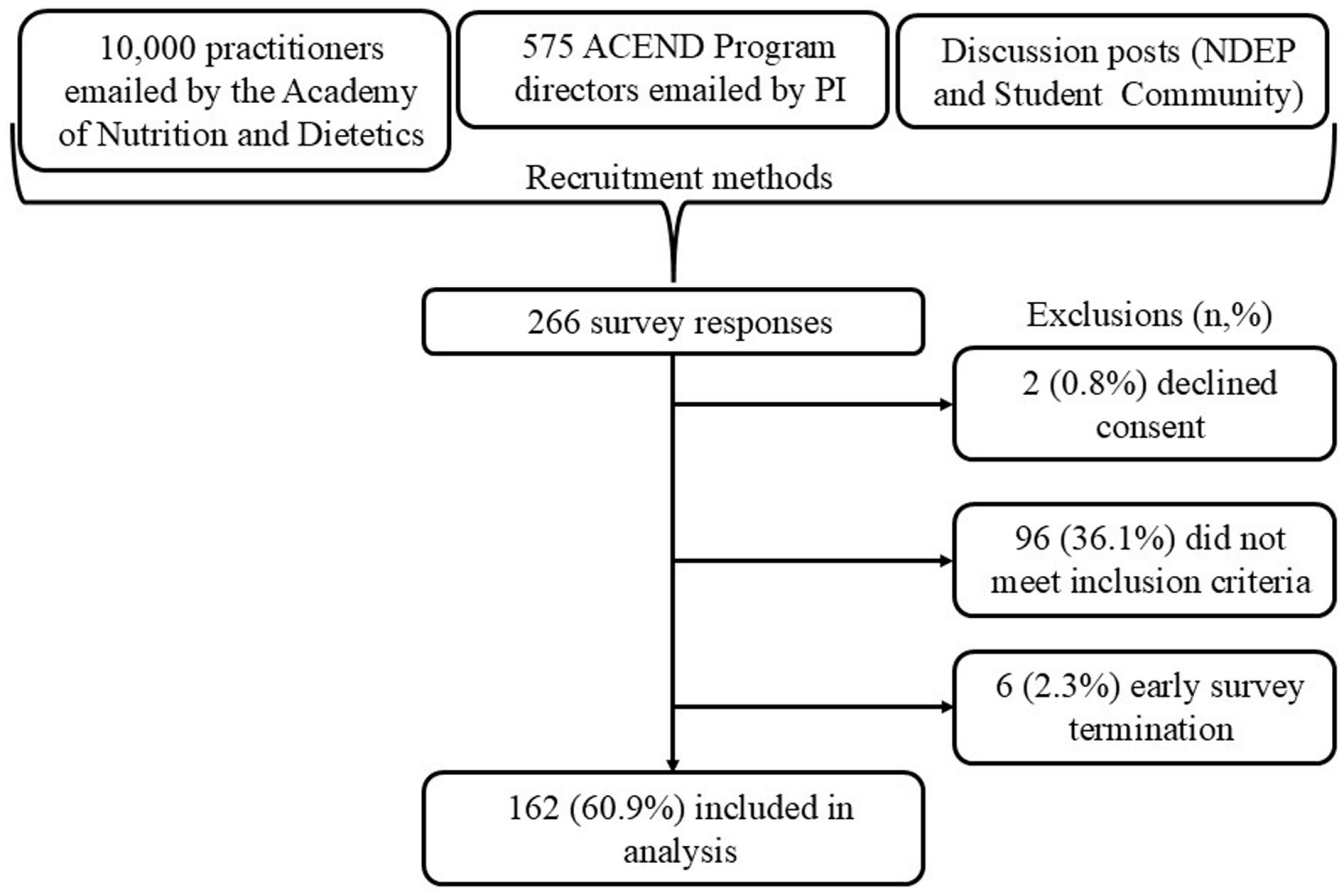
Recruitment and inclusion flow of study participants. A total of 266 survey responses were recorded following recruitment through professional outreach and community postings. Of these, 2 (0.8%) declined consent, 96 (36.1%) did not meet inclusion criteria, and 6 (2.3%) were incomplete (terminated early). A final sample of 162 participants was included in the analysis. ACEND, Accreditation Council for Education in Nutrition and Dietetics; PI, principal investigator; NDEP, nutrition and dietetics educators and preceptors.

A total of 266 survey responses were obtained, with participant flow detailed in Figure 1. Two participants were directed to the end of the study due to declining the consent statement. An additional 96 participants did not meet inclusion criteria due to non-alignment with the indicated populations. Six participants began but did not finish the survey. These observations were removed, as they appeared to be random in nature and were less than 5% of total responses. The attitude-related measures (variables A1-A16 and MAS) for one observation were negatively inclined and found to be outside of three standard deviations from the mean. These values for these variables were changed to match those of the next closest observation within the distribution.

Individual review of free-text data yielded three responses from variable D5, area of teaching/precepting, that were recoded to match the offered selections. Relevant “Other” responses from variables N4 and N5 are reported in Supplementary material.

### Descriptive analysis

A full review of the data yielded from descriptive analysis can be found in Table 1 and other Supplementary Tables S1–S4. The summary is shared in Table 1. Of the 162 participants, 89 (54.9%) reported as educators, 52 (32.1%) reported as preceptors, and 21 (13%) reported as being an educator and a preceptor (PE). The majority of RDN participants (136, 84%) reported having completed a graduate degree; however, terminal degrees were only strongly represented in the educator (*n* = 48, 53.9%) and PE (*n* = 6, 28.6%) categories. Only two preceptors (3.8%) reported possessing a terminal degree.

**Table 1:** Summary of participant characteristics.

Domain	Educator	Preceptor	Preceptor-educator	Total *n* (%)
Participant role	89 (54.9%)	52 (32.1%)	21 (13%)	162 (100%)
Graduate degree	83 (93.3%)	33 (63.5%)	20 (95.2%)	136 (84%)
Terminal degree	48 (53.9%)	2 (3.8%)	6 (28.6%)	56 (34.6%)
Year RDN requirements completed				
1975–1984	11 (12.4%)	2 (3.8%)	1 (4.8%)	14 (8.6%)
1985–1994	15 (16.9%)	4 (7.7%)	1 (4.8%)	20 (12.3%)
1995–2004	23 (25.8%)	11 (21.2%)	6 (28.6%)	40 (24.7%)
2005–2014	37 (41.6%)	21 (40.4%)	9 (42.9%)	67 (41.4%)
2015–2024	3 (3.4%)	14 (26.9%)	4 (19%)	21 (13%)
Education regional distribution				
South	31 (34.8%)	16 (30.8%)	9 (42.9%)	56 (34.6%)
Midwest	25 (28.1%)	16 (28.8%)	5 (23.8%)	46 (28.4%)
Northeast	19 (21.3%)	8 (15.4%)	2 (9.5%)	29 (17.9%)
West	14 (15.7%)	11 (21.2%)	3 (23.8%)	28 (17.3%)
Unspecified	—	1 (1.9%)	2 (9.5%)	3 (1.9%)
Practice regional distribution				
South	36 (40.4%)	18 (34.6%)	7 (33.3%)	61 (37.7%)
Midwest	13 (14.6%)	14 (26.9%)	6 (28.6%)	33 (20.4%)
Northeast	16 (18%)	7 (13.5%)	2 (9.5%)	25 (15.4%)
West	23 (25.8%)	12 (23.1%)	4 (19%)	39 (24.1%)
Unspecified	1 (1.1%)	1 (1.9%)	2 (9.5%)	4 (2.5%)
Practice setting/area instructed				
Inpatient care	25 (28.1%)	43 (82.7%)	13 (61.9%)	81 (50%)
Outpatient care	22 (24.7%)	44 (84.6%)	13 (61.9%)	79 (48.8%)
Research	30 (33.7%)	2 (3.8%)	4 (19%)	36 (22.2%)
Administrative/food service	10 (11.2%)	6 (11.5%)	2 (9.5%)	18 (11.1%)
Consultation/business practice	8 (9%)	5 (9.6%)	2 (9.5%)	15 (9.3%)
Public health/community	6 (6.7%)	5 (9.6%)	2 (9.5%)	13 (8%)
Academy of nutrition and dietetics membership	80 (89.9%)	30 (57.7%)	17 (80.1%)	127 (78.4%)
NCP/T implementation intention	79 (88.8%)	42 (80.8%)	20 (95.2%)	141 (87%)
Barriers to NCP/T implementation among non-implementers				
Belief that NCP/T does not apply to current role	4 (40%)	4 (40%)	—	8 (38.1%)
Lack of implementation confidence	—	2 (20%)	—	2 (9.5%)
Lack of management or institutional support	2 (20%)	3 (30%)	—	5 (23.8%)
Lack of motivation	2 (20%)	2 (20%)	—	4 (19%)
Lack of knowledge	—	1 (10%)	—	1 (4.8%)
Insufficient training resources/continuing education	1 (10%)	—	—	1 (4.8%)
Lack of time	1 (10%)	1 (10%)	—	2 (9.5%)
Lack of education and training	1 (10%)	1 (10%)	—	2 (9.5%)
Lack of financial resources	—	1 (10%)	1 (100%)	2 (9.5%)
Lack of peer support	1 (10%)	—	—	1 (4.8%)
Electronic health records unavailable	1 (10%)	—	1 (100%)	2 (9.5%)
Not having access to online tools or books	1 (10%)	—	—	1 (4.8%)
Other	1 (10%)	3 (30%)	—	4 (19%)
Any barrier reported	9 (90%)	10 (100%)	1 (100%)	20 (95.2%)
Sources of NCP/T education				
Didactic/supervised practice	49 (55.1%)	29 (55.8%)	9 (42.9%)	87 (53.7%)
Education sessions/oral presentations	35 (39.3%)	18 (34.6%)	5 (23.8%)	58 (35.8%)
Webinars/online modules	33 (37.1%)	16 (30.8%)	4 (19%)	53 (32.7%)
Workshops	27 (30.3%)	10 (19.2%)	3 (14.3%)	40 (24.6%)
Workplace resources/mentorship	29 (32.6%)	18 (34.6%)	5 (23.8%)	52 (32.1%)
Academy of nutrition and dietetics website	35 (39.3%)	20 (38.5%)	7 (33.3%)	62 (37.3%)
Dietetic associations/professional meetings	37 (41.6%)	9 (17.3%)	2 (9.5%)	48 (29.6%)
Perceived competence level				
Unfamiliar	2 (2.2%)	—	—	2 (1.2%)
Competent	14 (15.7%)	17 (32.7%)	7 (33.3%)	38 (23.5%)
Proficient	39 (43.8%)	23 (44.2%)	6 (28.5%)	68 (42%)
Mastery	34 (38.2%)	12 (23.1%)	8 (38.1%)	54 (33.3%)
Proficiency score mean (±SD)	2.4 (0.77)	2.06 (0.8)	2.43 (0.68)	2.30 (0.78)
Mean attitude score mean (±SD)	3.89 (0.73)	3.38 (0.84)	3.42 (1.1)	3.68 (0.83)

NCP, nutrition care process; NCP/T, nutrition care process and nutrition care process terminology.

Assessment of the year RDN requirements were completed demonstrates that over half of participants (*n* = 88, 54.3%) were registered 2005 or later and likely received some degree of NCP/T training during or prior to registration examination. Practitioners completing requirements within the years 2005–2014 (*n* = 67, 41.4%) were most strongly represented, whereas those finishing the process in 1975-1974 (*n* = 14, 8.6%) were least represented.

Distribution of participants’ location (by state and region) of education varied. Of the 50 states, 42 were represented. Responses from Alaska, Hawaii, Maine, Nevada, New Hampshire, Vermont, West Virginia, and Wyoming were not recorded. Furthermore, states such as Delaware, Indiana, Kansas, Kentucky, Louisiana, Montana, Nebraska, Oregon, South Carolina, South Dakota, and Virginia remained poorly represented with only one observation from each state. Illinois (*n* = 16, 9.9%), California (*n* = 14, 8.6%), Texas (*n* = 14, 8.6%), and New York (*n* = 13, 8%) were the states with the strongest representation. The United States Midwest (*n* = 46, 28.4%) and United States South regions (*n* = 56, 34.6%) were most prominent, with RDNs from the United States Northeast (*n* = 29, 17.9%) and United States West (*n* = 28, 17.3%) demonstrating less representation.

Despite small variances in state data, the participants’ location of practice was similar to location of education completion by region. Alaska, Colorado, Connecticut, Delaware, Hawaii, Idaho, Kansas, Maine, New Hampshire, New Mexico, South Dakota, and Wyoming were not in the collected data. Arizona, Kentucky, Montana, Nebraska, Nevada, North Dakota, Oregon, Vermont, and Virginia were similarly poorly represented with one observation from each state. The regional distribution remained similar, with the United States South (*n* = 61, 37.7%) leading representation. In descending order of representation, the United States South was followed by the United States West (*n* = 39, 24.1%), the United States Midwest (*n* = 33, 20.4%), and the United States Northeast (*n* = 25, 15.4%).

Patient care settings (inpatients and outpatients) were most frequently reported by participants of all categories, with 81 (50%) participants reporting involvement in inpatient dietetics and 79 (48.8%) participants involved in outpatient care. Educators (*n* = 30, 33.7%) and PEs (*n* = 4, 19%) demonstrated notably higher involvement in research than those reporting to be preceptors (*n* = 2, 3.8%). Regardless of category, administrative food service, consultation and business practice, and public health settings were among the least frequently reported.

Reported Academy membership varied by category, with preceptors (*n* = 30, 57.7%) displaying the lowest membership rates. Educators and PEs displayed high rates (*n* = 80, 89.9% and *n* = 17, 80.1%, respectively) of membership.

Collectively, 141 participants (87%) reported current implementation or intention to implement the NCP/T. One PE withheld answering the item and was not included in this analysis. Preceptors (*n* = 42, 80.8%) reported the lowest rates. PEs (*n* = 20, 95.2%) reported a higher implementation rate than educators (*n* = 79, 88.8%).

Of the 21 participants reported non-implementation, 20 indicated barriers encountered. The most prominently reported barriers were reported by educators and preceptors and included the belief that NCP/T does not apply to their work (*n* = 8, 38.1%), lack of management support (*n* = 5. 23.8%), and lack of motivation (*n* = 4, 19%). Free text responses reported as “Other (please specify)” further support these observations and can be reviewed in Supplementary material.

Lessons encountered in the didactic and/or supervised practice component are suggested to be among the largest and most influential sources of education regardless of category, as 87 (53.7%) of all participants reported this option. Other well-established inter-category sources of education include education sessions with oral presentations (*n* = 58, 35.8% of participants), workshops (*n* = 40, 24.6% of participants), and webinars (*n* = 53, 32.7% of participants), workplace resources (*n* = 52, 32.1%), and the Academy website (*n* = 62, 37.3% of participants). Content from dietetic associations displayed the largest difference between categories, which educators reported higher frequency (*n* = 37, 36.3%) than preceptors (*n* = 9, 17.3%) and PE (*n* = 2, 9.5%).

Nearly all participants (*n* = 160, 98.7%) reported perceptions of having baseline competence with NCP/T usage. Preceptors and PEs reported similar percentages as it relates to being perceived as competent (*n* = 17, 32.7% and *n* = 7, 33.3% respectively). Educators demonstrated the lowest frequency of being perceived as competent, with 14 (15.7% of educators) selecting this level. Educators and preceptors reported similar percentages of their individual categories for proficiency (*n* = 39, 43.8% and *n* = 23, 44.2% respectively). Six PE (28.5% of this category) perceived themselves to be proficient. With almost identical group percentages, 34 educators (38.2%) and eight PE (38.1%) indicated perceived utilization of the NCP/T with mastery. Preceptors had the lowest frequency (*n* = 12, 23.1%).

**Figure 2:**
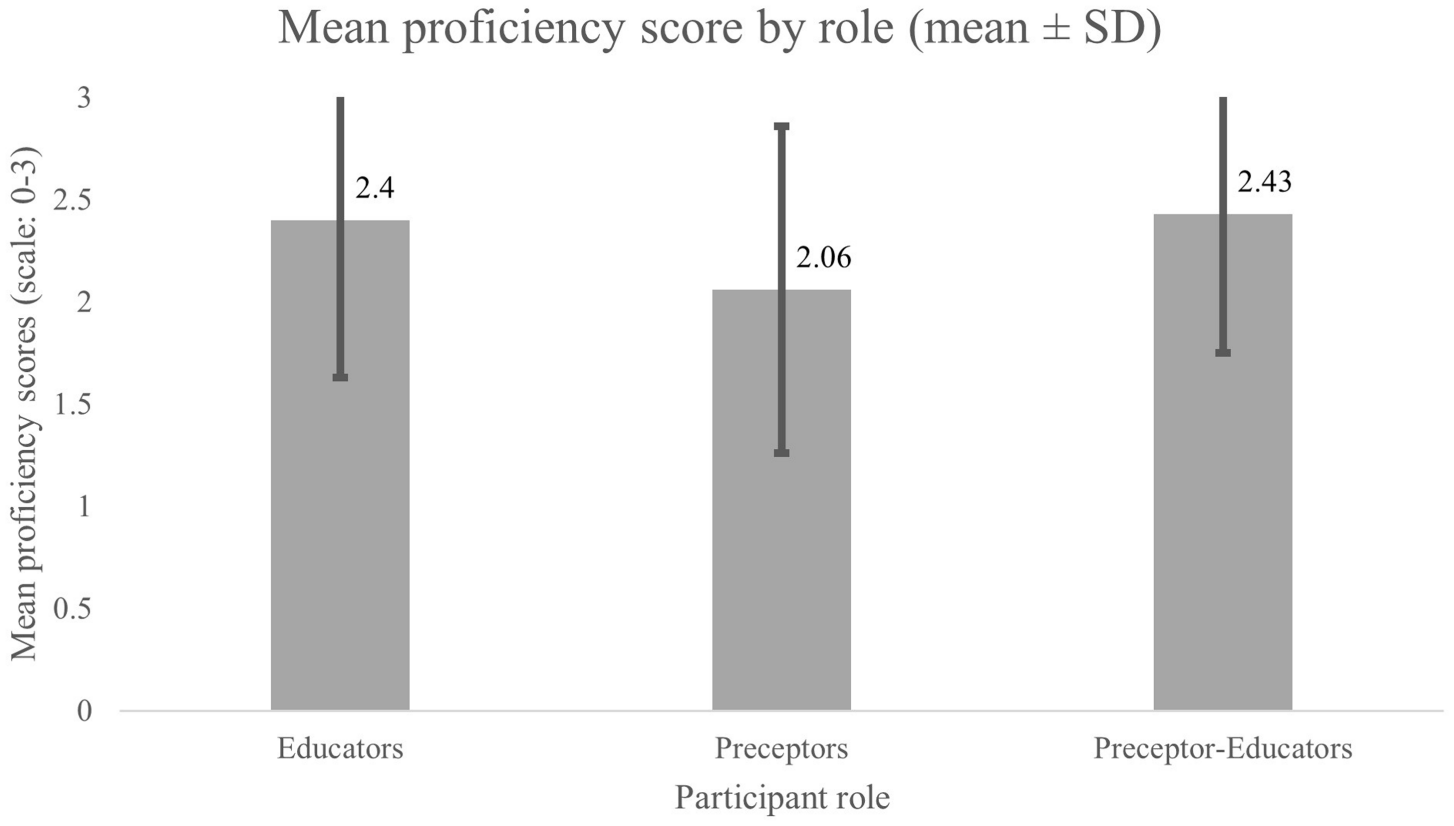
Mean proficiency score by role (mean ± SD). Mean proficiency scores (scale: 0–3) are shown for educators, preceptors, and preceptor–educators. Error bars represent standard deviation. Preceptors demonstrated lower mean proficiency scores compared with educators and preceptor–educators.

**Figure 3:**
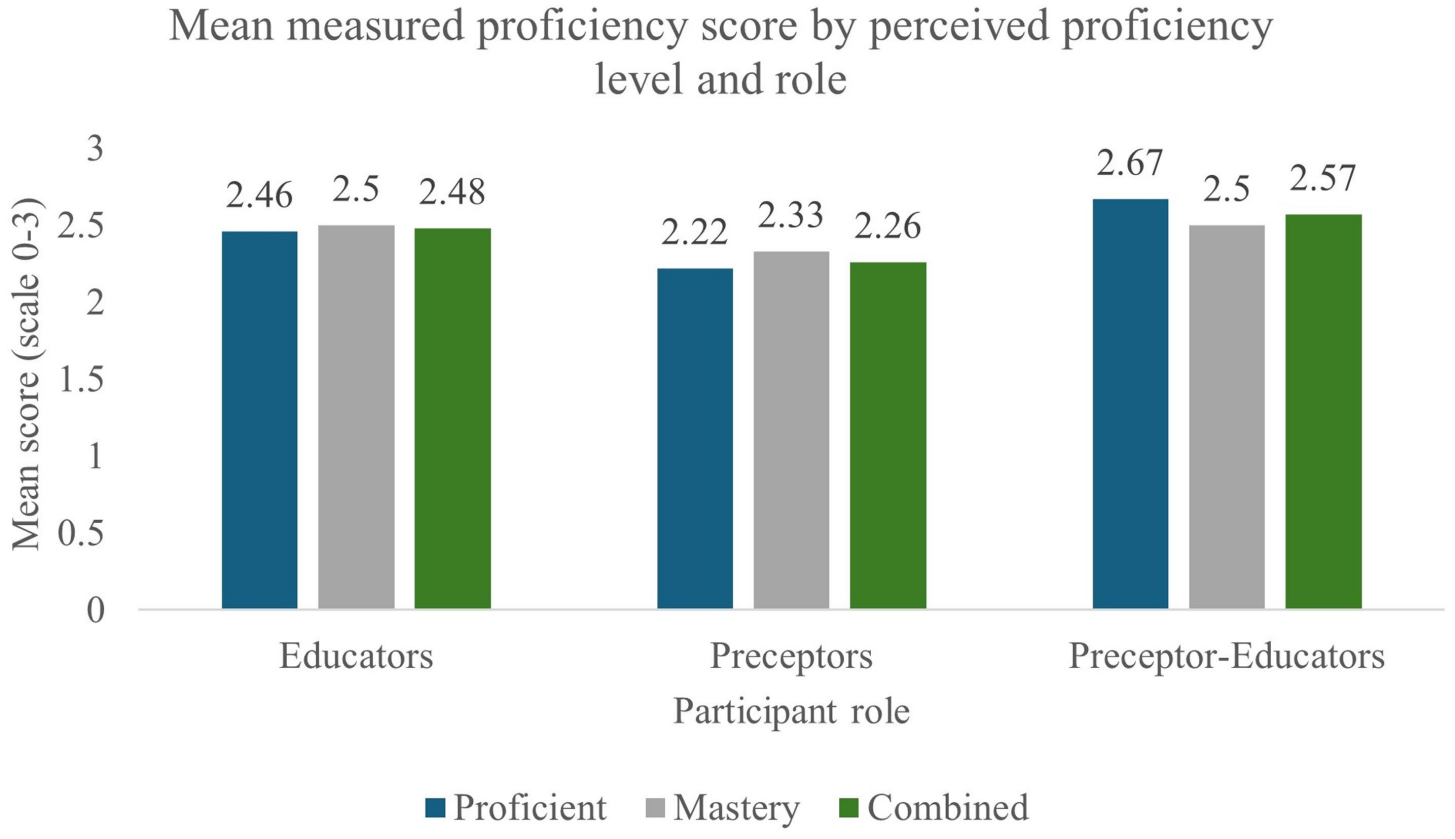
Mean measured proficiency score by perceived proficiency level and role. Mean proficiency scores (scale: 0–3) are presented for educators, preceptors, and preceptor–educators across perceived proficiency categories (proficient, mastery, and combined). Scores reflect assessed proficiency (PS) within each subgroup. Overall, preceptors demonstrated lower mean proficiency scores across categories, whereas preceptor–educators exhibited the highest scores. Differences illustrate variation in alignment between perceived and measured proficiency across roles.

Differences between groups for each proficiency item as well as proficiency scores (PS) can be reviewed in Table 2. The mean PS across all categories was 2.3. Educators and preceptors demonstrated higher mean scores, as displayed in Figure 2. When evaluating means, educators and PE consistently demonstrated higher scores across all items. The mentioned pattern can also be seen when evaluating scores by perceived proficiency. Comparing means, educator and PE PS exceeded those of preceptors at every perceived competence level (except for “Unfamiliar,” which did not have preceptor or PE representation). Preceptor mean scores across the proficient, mastery, and combined proficient–mastery levels were lower than those of educators (Figure 3, Supplementary Figure 1). Additionally, educator and PE mean scores at the proficient level outperformed preceptors at every level (including mastery).

**Table 2:** Proficiency item scores by category.

Item	Educator (*n* = 89)		Preceptor (*n* = 52)		Preceptor-educator (*n* = 21)	
	Mean (SD)	Median (IQR)	Mean (SD)	Median (IQR)	Mean (SD)	Median (IQR)
P1: By theory, proper implementation of the NCP/T should result in a reduction of which of the following care-related aspects?	0.84 (0.37)	1 (1)	0.71 (0.46)	1 (1)	0.76 (0.44)	1 (1)
P2: Which of the following is the first concept in the NCP chain?	0.69 (0.47)	1 (1)	0.48 (0.51)	0 (1)	0.67 (0.48)	1 (1)
P3: As a monitoring indicator, documented changes in patient/client weight would be best placed in which of the following categories during development of a PES statement?	0.88 (0.33)	1 (1)	0.87 (0.35)	1 (1)	1 (0)	1 (0)
Proficiency score	2.4 (0.77)	3 (1)	2.06 (0.8)	2 (2)	2.43 (0.68)	3 (1)

NCP, nutrition care process; NCP/T, nutrition care process and nutrition care process terminology; PES, problem-etiology-symptoms.

**Figure 4:**
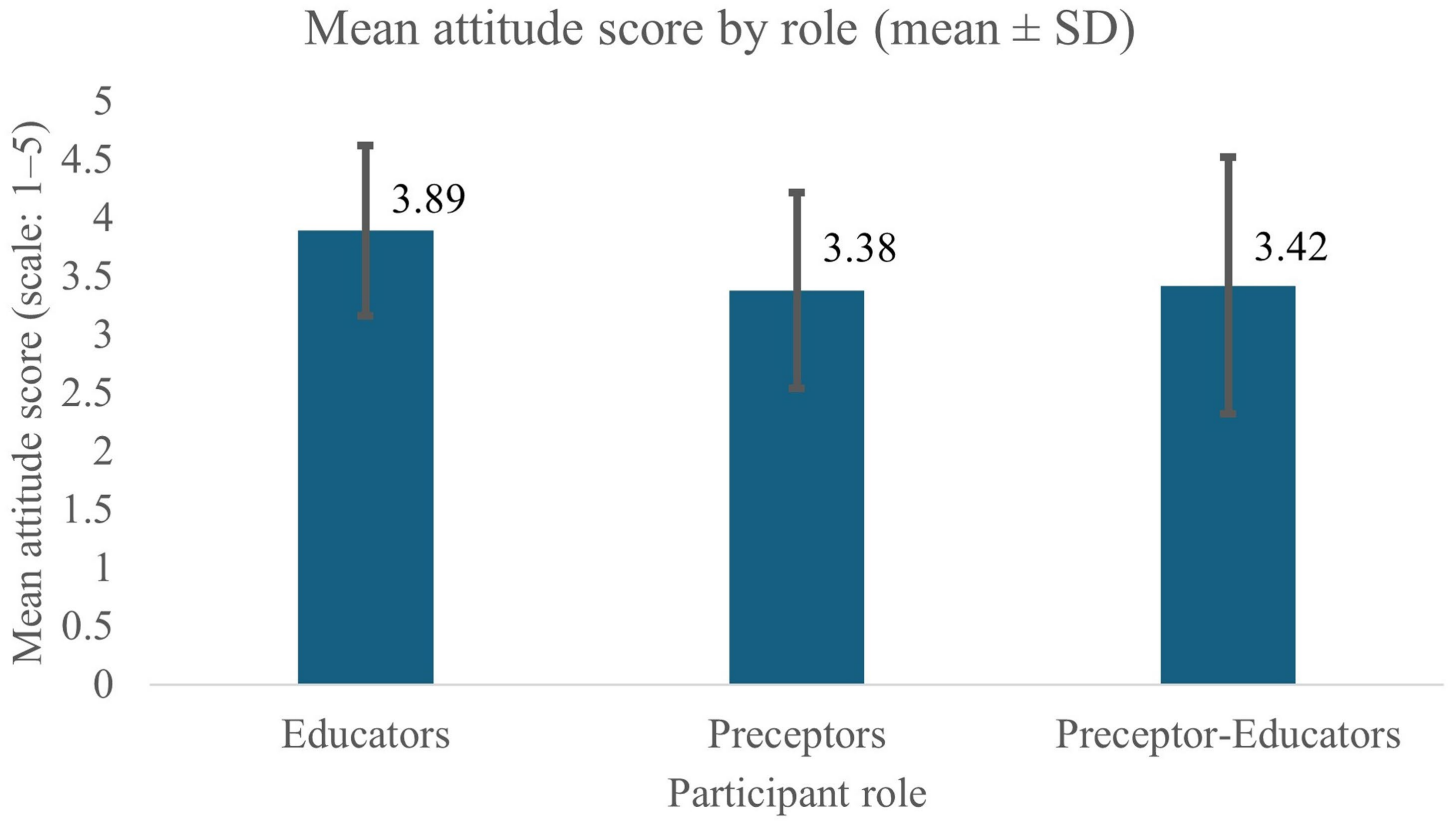
Mean attitude score by role (mean ± SD). Mean attitude scores (scale: 1–5) are presented for educators, preceptors, and preceptor–educators. Error bars represent standard deviation. Educators demonstrated the highest mean attitude scores, whereas preceptors exhibited lower scores, with preceptor–educators showing intermediate values.

The mean for all participants’ mean attitude scores (MAS) was 3.68 (0.83). Measures of central tendency suggest a neutral-to-positive attitude toward NCP/T across all groups. Baseline review indicated that the highest MAS were reported by educators, as seen in Figure 4. This is followed by PE and preceptors, with the latter reporting the lowest MAS. Assessment of the lowest (A4, A9, A12, and A13) and highest quartile items (A1, A5, A6, and A14) suggest that participants felt more negatively about the relationship between the NCP/T and communication with recipients of care and other disciplines, and more positively about the structure, organization, and teaching capabilities associated with NCP/T. All item scores and MAS by category can be reviewed in Table 3.

**Table 3:** Attitude item scores by category.

Item	Educator (*n* = 89)		Preceptor (*n* = 52)		Preceptor-educator (*n* = 21)	
	Mean (SD)	Median (IQR)	Mean (SD)	Median (IQR)	Mean (SD)	Median (IQR)
A1: There are benefits to implementing the NCP (the process).	4.2 (0.87)	4 (1)	3.67 (0.97)	4 (1)	3.48 (1.44)	4 (2)
A2: There are benefits to implementing the NCPT (the terminology).	4.09 (0.86)	4 (1)	3.57 (1.12)	4 (1)	3.71 (1.15)	4 (1)
A3: The NCP and NCPT allow for clearer documentation.	4.08 (0.93)	4 (1)	3.44 (1.27)	4 (1)	3.57 (1.43)	4 (3)
A4: The NCP and NCPT help dietitians to become recognized as more valuable members of interprofessional health-care teams.	3.37 (1.11)	3 (1)	2.73 (1.17)	3 (2)	3.05 (1.32)	3 (2)
A5: The NCP provides dietitians with a consistent structure and framework for nutrition care.	4.15 (0.94)	4 (1)	3.81 (0.95)	4 (1)	3.76 (1.18)	4 (2)
A6: The NCPT provides dietitians with a common vocabulary to identify nutrition problems.	4.42 (0.72)	5 (1)	3.83 (1)	4 (0)	4.05 (1.07)	4 (1)
A7 The NCP and NCPT allow for more consistent care when patients/clients are transferred to other care settings.	3.99 (0.89)	4 (2)	3.44 (1.11)	4 (1)	3.67 (1.32)	4 (2)
A8: The NCP and NCPT facilitate communication between dietitians.	4.09 (0.87)	4 (1)	3.63 (0.97)	4 (1)	3.33 (1.43)	4 (3)
A9: The NCP and NCPT facilitate communication with other health-care professionals.	3.42 (0.17)	4 (2)	2.85 (1.16)	3 (2)	2.95 (1.47)	3 (3)
A10: The NCP and NCPT improve patient/client nutrition care.	3.85 (0.89)	4 (2)	3.48 (1.06)	4 (1)	3.52 (1.37)	4 (2)
A11: The NCP and NCPT encourage critical thinking among dietitians.	4.06 (0.86)	4 (1)	3.44 (1.21)	4 (1)	3.24 (1.41)	3 (3)
A12: The NCP and NCPT facilitate more patient/client involvement in the care process.	3.18 (0.96)	3 (2)	2.82 (0.97)	3 (2)	2.57 (1.21)	2 (1)
A13: The NCP and NCPT allow for a holistic perspective on the patients’/client’s situation.	3.51 (1.04)	4 (1)	3.02 (1)	3 (2)	3.10 (1.38)	4 (3)
A14: The NCP and NCPT help with training of dietetic students/interns during internship/practice placements.	4.21 (0.91)	4 (1)	3.62 (1.09)	4 (1)	3.90 (1.18)	4 (1)
A15: The NCP and NCPT support research on patient/client outcomes.	3.83 (1)	4 (2)	3.38 (0.99)	3 (1)	3.52 (1.47)	4 (3)
A16: The NCP and NCPT support evaluation and development of dietetic practice at the organizational level.	3.81 (0.9)	4 (1)	3.37 (1.12)	4 (1)	3.52 (1.37)	4 (2)
Mean attitude score	3.89 (0.73)	3.94 (1)	3.38 (0.84)	3.63 (1)	3.42 (1.10)	3.88 (2)

NCP, nutrition care process; NCP/T, nutrition care process and nutrition care process terminology.

### Inferential analysis

Chi-square evaluation of nominal and ordinal variables indicated that education level, Academy membership, and perceived NCP competency had significant relationships with NCP/T implementation intention. Effect sizes ranged from small-to-medium, with perceived NCP competence demonstrating the highest effect size and Academy membership as the lowest. Test values can be found in Table 4.

**Table 4:** Summary of Chi-square tests of association with nutrition care process implementation intention.

Variable	*χ*^2^ (d*f*)	*p*	*V*/*φ*
D2: Category	3.24 (2)	0.198	0.142
D3: Education level	18.26 (2)	<0.001*	0.337
D4: Year education completed	36.69 (43)	0.740	0.477
D5: Teaching area	14.76 (8)	0.064	0.195
D6: State educated	28.26 (38)	0.875	0.420
D6R: Region educated	1.84 (4)	0.766	0.107
D7: State practicing	27.71 (37)	0.866	0.416
D7R: Region practicing	1.50 (4)	0.827	0.096
D8: Academy member	10.18 (1)	0.001*	0.251
N2: Perceived competency	30.84 (3)	<0.001*	0.438
N5: NCP/T education origin	5.75 (12)	0.928	0.108
N6: Country trained if outside United States	0.15 (1)	0.698	0.031

*Significance threshold set at *p* < 0.05.

NCP/T, nutrition care process and nutrition care process terminology.

Mann–Whitney *U* tests were used during inferential analysis of PS and MAS due to parametric assumption violations. The distributions of MAS and PS were found to be abnormal, as evidenced by histogram review and remarkable Kolmogorov–Smirnov (*p* = 0.009 and *p* < 0.001 respectively) and Shapiro–Wilk tests (*p* < 0.001 for both variables). Levene’s test also indicated inequality in variance (*p* = 0.015) in MAS, though the output remained nonsignificant for PS (*p* = 0.786).

PS and MAS were both found to have significant relationships with NCP/T implementation intention, demonstrating small-to-medium effect sizes. All proficiency and attitude items except for P1, P3, Q4, Q9, Q12, Q15, and Q16 demonstrated significant relationships with NCP/T implementation. The analysis for item P3 was borderline significant, *p* = 0.05. Test results can be viewed fully in Table 5.

**Table 5:** Summary of Mann–Whitney *U* tests of continuous predictors by nutrition care process implementation intention.

Variable	*U*	*Z*	*p*	*R*
P1: By theory, proper implementation of the NCP/T should result in a reduction of which of the following care-related aspects?	1676.5	1.466	0.143	0.116
P2: Which of the following is the first concept in the NCP chain?	1865.5	2.355	0.019*	0.186
P3: As a monitoring indicator, documented changes in patient/client weight would be best placed in which of the following categories during development of a PES statement?	1683.5	1.963	0.05*	0.155
A1: There are benefits to implementing the NCP (the process).	1863	2.577	0.01*	0.204
A2: There are benefits to implementing the NCPT (the terminology).	1851.5	2.520	0.012*	0.199
A3: The NCP and NCPT allow for clearer documentation.	2164	3.662	<0.001*	0.289
A4: The NCP and NCPT help dietitians to become recognized as more valuable members of interprofessional health-care teams.	1838.5	1.905	0.057	0.150
A5: The NCP provides dietitians with a consistent structure and framework for nutrition care.	1903.5	2.325	0.02*	0.183
A6: The NCPT provides dietitians with a common vocabulary to identify nutrition problems.	1981.5	2.825	0.005*	0.223
A7 The NCP and NCPT allow for more consistent care when patients/clients are transferred to other care settings.	1859.5	2.046	0.041*	0.161
A8: The NCP and NCPT facilitate communication between dietitians.	1901	2.288	0.022*	0.180
A9: The NCP and NCPT facilitate communication with other health-care professionals.	1615.5	0.750	0.453	0.059
A10: The NCP and NCPT improve patient/client nutrition care.	1947.5	2.513	0.012*	0.198
A11: The NCP and NCPT encourage critical thinking among dietitians.	1948.5	2.518	0.012*	0.198
A12: The NCP and NCPT facilitate more patient/client involvement in the care process.	1718.5	1.367	0.172	0.108
A13: The NCP and NCPT allow for a holistic perspective on the patients’/client’s situation.	1905.5	2.268	0.023*	0.179
A14: The NCP and NCPT help with training of dietetic students/interns during internship/practice placements.	2014	2.908	0.004*	0.229
A15: The NCP and NCPT support research on patient/client outcomes.	1750.5	1.467	0.142	0.116
A16: The NCP and NCPT support evaluation and development of dietetic practice at the organizational level.	1736.5	1.408	0.159	0.111
Proficiency score	1998.5	2.890	0.004*	0.228
Mean attitude score	2013.5	2.801	0.005*	0.221

*Significance threshold set at *p* < 0.05.

NCP, nutrition care process; NCP/T, nutrition care process and nutrition care process terminology.

## Discussion

### Attitudes, implementation, and professional role

Most participants reported either current implementation or an intention to implement the NCP/T (86.4%). Although these implementation rates exceed those reported in generalized international cohorts (26, 27), the findings align with expectations. This alignment is largely attributable to the 2017 INIS distribution (27) which documented high NCP/T implementation scores after adjustment for country, education, years since training, and other practice areas among RDNs in direct patient care roles and academic educators—the primary groups represented in this sample. Implementation rates differed across educator type, with preceptors reporting the lowest implementation rate and preceptor-educators the highest. Though not a component of the original objectives of the investigation, follow-up Chi-square analyses were unable to find a statistically significant difference between categories, *χ*^2^ (2) = 5.44, *p* = 0.142. Further assessment of implementation across education-role subcategories is warranted, as no prior investigations have evaluated these differences to our knowledge.

MAS, a variable of significant interest, scores were neutral-to-positive overall, which continues the trends observed in previous investigations (17, 26). Notably, preceptors consistently reported lower attitudes than educators and preceptor-educators. Non-objective oriented Kruskal–Wallis analysis revealed significant differences in MAS among role categories (*H*^2^ = 12.06; *p* = 0.002). *Post hoc* analyses using Dunn’s procedure with Bonferroni correction indicated that educators scored significantly higher than preceptors (*p* = 0.0019). The lower MAS scores in the preceptor population partially supports the observed decrease in implementation rates; although, more thorough investigation of variables with a larger sample is indicated.

Similar patterns were observed with further analysis secondary to the original objectives. PS values were higher among educators and preceptor-educators than among preceptors. Significant difference between PS across groups *H*^2^ = 7.84, *p* = 0.02, with *post hoc* pairwise comparisons using Dunn’s procedure with Bonferroni correction demonstrated that educators reported significantly higher PS than preceptors (*p* = 0.039). These differences may be partially explained through several structural characteristics. Follow up Chi-square analyses indicated that significant differences in degree acquisition between categories were present, (*χ*^2^ (4) = 48.34; *p* < 0.001). Examination of the data suggests that educators were more likely to hold doctoral degrees, whereas preceptors were more likely to hold bachelor’s and less likely to hold doctorates.

Education levels among participants with combined educator/preceptor roles did not differ significantly from expected frequencies, suggesting that RDNs in educational roles were more likely to hold advanced degrees. While there is no evidence known to these authors that examines degree level and NCP/T competence, a follow-up chi-square test of independence was conducted. Results demonstrated a significant association between highest degree earned and proficiency score (*χ*^2^ (6) = 19.13, *p* = 0.004), with higher proficiency scores more frequently observed among respondents with master’s and doctoral degrees compared with those holding bachelor’s degrees. Collectively, these findings trend with INIS data, as higher degree status has been previously associated with NCPT implementation (26, 27). Proposed updates to the 2027 ACEND accreditation standards, now requiring directors of various graduate-level dietetics programs to obtain a terminal degree, and the increased availability of degree options for RDNs in education may continue to ensure maintenance or growth of NCP/T comprehension in this group (28); however, this is likely to disproportionately increase the gap between those in preceptor roles.

Similar themes in data were also observed with Academy membership. Additional Chi-square analyses revealed a significant association between professional role and Academy membership (*χ*^2^ (2) = 20.18; *p* < 0.001), with educators being more likely to be Academy members and preceptors being less likely to have membership. Membership rates among participants with combined educator/preceptor roles were consistent with expected distributions. Across international and national studies evaluating NCP/T implementation, professional or Academy membership has not been examined as an individual-level predictor of implementation or knowledge. Although dietetic associations are frequently described as coordinating implementation strategies and providing access to resources, membership itself has not been empirically evaluated in relation to NCP/T use (26, 27). By extension of previous studies and our findings, Academy membership may represent a potential mechanism for addressing common barriers to NCP/T implementation—such as access to resources (computer software, workplace implementation resources, and training materials), motivation, and peer support. Continued analysis of region-specific INIS data support this inference, as multiple barriers encountered in France and Germany would be influenced by these benefits associated with Academy membership (14). Follow-up investigations within the United States are warranted to continue to validate these observations.

Each of these characteristics demonstrated a significant relationship with implementation intention, and their concurrent presence among educators may help explain the comparatively higher implementation prevalence in this group. Taken together, the alignment of significantly lower attitudes, proficiency, education level, and Academy membership suggests that preceptors may represent an implementation-vulnerable population. Since preceptors directly influence how students apply the NCP/T in clinical settings, reduced use among this group introduces a critical point of vulnerability in pre-registration training. Students often model the behaviors they observe most frequently; thus, lower preceptor implementation risks encouraging inconsistent evidence-based practices and weakening long-term NCP/T adoption across the discipline. Addressing this gap through targeted preceptor development may be essential for sustained, national implementation.

### Proficiency and its relationship to implementation

Mean proficiency scores exceeded mid-range values across groups, with educators and preceptor-educators consistently outperforming preceptors across items and across all perceived competence levels. Both MAS and PS demonstrated significant associations with implementation intention, each with small-to-medium effect sizes. These observations align with prior clinical investigations indicating that knowledge, perceived competence, and standard-based reasoning are foundational qualities for routine application of care models such as the NCP/T (8, 15, 17, 29, 30). While causality cannot be inferred, the overlap of decreases in MAS, PS, and implementation intention among preceptors introduces a notable pattern.

### Contextualization of reported barriers within these findings

Among non-implementers, the most frequently reported barriers included lack of management support, limited motivation, and the belief that the NCP/T does not apply to their work. These barriers have been described in prior samples (14–16, 27) and were again observed in this investigation. These findings are consistent with a recent mixed-methods investigation of United States-based RDNs that identified these three barriers among the key determinants of NCP/T use (16). That analysis further highlighted the importance of system-level enablers, such as institutional expectations and access to resources, in supporting implementation. The present findings extend this work by demonstrating that similar barriers are differentially distributed across educational roles, with preceptors appearing particularly vulnerable to non-implementation.

The belief that the NCP/T does not apply was disproportionately reported by those practicing in outpatient settings, and outpatient preceptors appeared to endorse this belief more frequently than outpatient educators. This same subgroup also demonstrated lower mean proficiency scores than the overall pool of non-implementers in the sample, suggesting that role-and-context–specific perceptions of applicability may co-occur with lower knowledge or confidence.

Although causality cannot be established, the convergence of lower implementation prevalence among preceptors in the present study, recurrence of the “does not apply” barrier, and prior evidence indicating that outpatient preceptors are both more likely to hold this belief and to show comparatively lower proficiency, suggests that outpatient preceptors may represent a population of particular interest for future investigation. Further investigation is warranted in this population, as contextual factors (such as reduced patient clinical acuity, variable workloads, and responsibilities beyond direct patient care) may differentially influence NCP/T implementation and its determinants compared with other practice settings.

Comparable implementation patterns have been documented across other health professions. In nursing, physical therapy, occupational therapy, and primary care medicine, non-adoption of evidence-based practices or standardized guidelines is frequently attributed to similar trends identified in dietetics literature and this study. These include limited organizational support and resources, low priority or motivation, and perceived lack of applicability to local practice contexts or patient populations (31–36). While these interdisciplinary studies pertain to various aspects of evidence-based care as opposed to care models and terminology, the findings suggest these barriers as critical areas of intervention for outpatient preceptors. The recurrence of the same barrier structure across studies, despite differences in design and sampling, further reinforces the interpretive stability of this observation.

### Implications

The present findings have two primary implications for future methodological and educational work. First, the observation of associations between MAS, PS, and implementation may inform refinement of existing attitude instruments. Many individual attitude items were significantly associated with implementation intention, but several were not. If this pattern persists across other deployments, an abbreviated attitude measure—retaining only items empirically related to implementation—may be feasible. Such a tool could enhance efficiency in future deployments and reduce measurement redundancy, particularly if developed using item-level evidence from this sample in combination with existing INIS datasets (19).

At the practice and training level, the findings reinforce the enduring influence of pre-registration education, as more than half of participants reported first encountering the NCP/T in didactic or supervised practice settings. This supports prior findings, such as those from Desroches and colleagues (17), suggesting that foundational exposure during educational programming may shape subsequent attitudes and behaviors. The recurring identification of outpatient preceptors as a subgroup with lower proficiency and more frequent endorsement of barriers suggests that this population may be particularly susceptible to educational intervention. Because preceptors directly model normal practice behaviors to students, differences in preceptor attitudes and NCP/T usage may indirectly influence the next generation of practitioners through observational learning, feedback, and expectation setting. If future work confirms that preceptor-level characteristics influence trainees’ adoption, intervention in this area could have substantial effects even if targeted at a relatively small subgroup. These results support the need for targeted preceptor development initiatives—such as structured training modules focused on NCP/T application, competency reinforcement, and barrier mitigation. At the program level, integrating standardized NCP/T reinforcement strategies into both didactic curricula and supervised practice requirements may further support consistent adoption.

These implications emphasize the value of distinguishing between structural and perceptual determinants of implementation in future evaluations. At the systems level, integration of NCP workflows into electronic health records and institutional protocols may facilitate more consistent application. Additionally, professional organizations and accrediting bodies may play a key role in promoting standardized implementation through policy development, competency requirements, and curriculum alignment. However, these inferences should be interpreted in the context of the study sample, and future research is needed to determine whether these patterns generalize across more diverse and nationally representative populations.

### Limitations

This investigation has multiple limitations that should be considered when interpreting the findings. Despite a robust sampling strategy, the study did not reach the target sample size required for optimal statistical power. An *a priori* power analysis indicated a target of 370 participants; however, only 162 respondents qualified and completed the survey. This shortfall was partially influenced by the application of exclusion criteria, which removed 104 individuals (39.1%) who initially began the survey. The reduced sample size may have limited the ability to detect small-to-moderate relationships among variables, increasing the risk of Type II error. Furthermore, the smaller-than-intended sample may affect the stability of psychometric estimates and limit the robustness of subgroup analyses. Accordingly, caution is warranted when generalizing these findings to the broader population of registered dietitian nutritionists and dietetics trainees.

Beyond overall sample size, the distribution of participants across professional roles was uneven, with educators comprising a disproportionately large segment relative to preceptors and preceptor-educators. This imbalance influenced the selection of statistical approaches and may reduce the precision and interpretability of role-based comparisons. Geographic representation was also limited. Several states were not represented, and many contributed only a single observation, restricting the national representativeness of the sample. This uneven distribution introduces the potential for regional bias, as educational preparation, clinical practice patterns, and access to professional development opportunities may vary across geographic contexts. As a result, the findings may disproportionately reflect the experiences of individuals from more heavily represented regions.

Additional sources of bias are inherent to the data collection methodology. All variables were assessed via self-report, introducing the potential for response inflation and social desirability bias, particularly for constructs related to perceived competence and behavioral intention. Moreover, recruitment relied on electronic dissemination and voluntary participation, which increases the risk of nonresponse bias. Individuals with greater interest, familiarity, or engagement with the Nutrition Care Process and Terminology may have been more likely to participate, potentially influencing the observed results.

## Conclusion

Most educators, preceptors, and preceptor-educators in this sample reported implementation or intention to implement the NCP/T, with educators and preceptor-educators demonstrating the highest reported rates. Attitudes and proficiency were each associated with implementation intention, and both were comparatively lower among preceptors. These findings parallel prior reports, suggesting that implementation variation across roles persists even two decades after the formal adoption of the NCP/T.

Despite high overall implementation, several recurring barriers were identified among non-implementers, including lack of management support, limited motivation, and the belief that the NCP/T does not apply to their work. The recurrence of these barriers across investigations, coupled with the observed distribution of attitudes and proficiency, suggests that implementation resistance may be driven less by structural inapplicability and more by uneven distribution of enabling determinants across practice settings and roles.

These findings are consistent with the interpretation that variation in NCP/T implementation reflects differences in determinants beyond simple awareness or exposure. Role-based differences in attitudes, proficiency, and contextual support appear to coincide with lower implementation rates, particularly among preceptors. Additional investigation in larger and more balanced samples is warranted to confirm these patterns and to further examine the extent to which modifiable determinants contribute to persistent heterogeneity in NCP/T use across the profession.

## Data Availability

The raw data supporting the conclusions of this article will be made available by the authors, without undue reservation.

## Supplementary material

SUPPLEMENTARY FIGURE 1 Median measured proficiency score by perceived proficiency level and role. Median proficiency scores (scale: 0–3) are shown for educators, preceptors, and preceptor–educators across perceived proficiency categories (proficient, mastery, and combined). Educators demonstrated uniformly high median scores, suggesting a ceiling effect, whereas preceptors showed lower and more variable scores. Preceptor–educators exhibited intermediate patterns.
