# Supplementary figures and images for: Factors influencing nutrition care process and nutrition care process terminology implementation among United States dietetics educators and preceptors
Source: Front Nutr. 2026 Apr 29;13:1805075. doi: 10.3389/fnut.2026.1805075 (PMC13167533; doi:10.3389/fnut.2026.1805075)

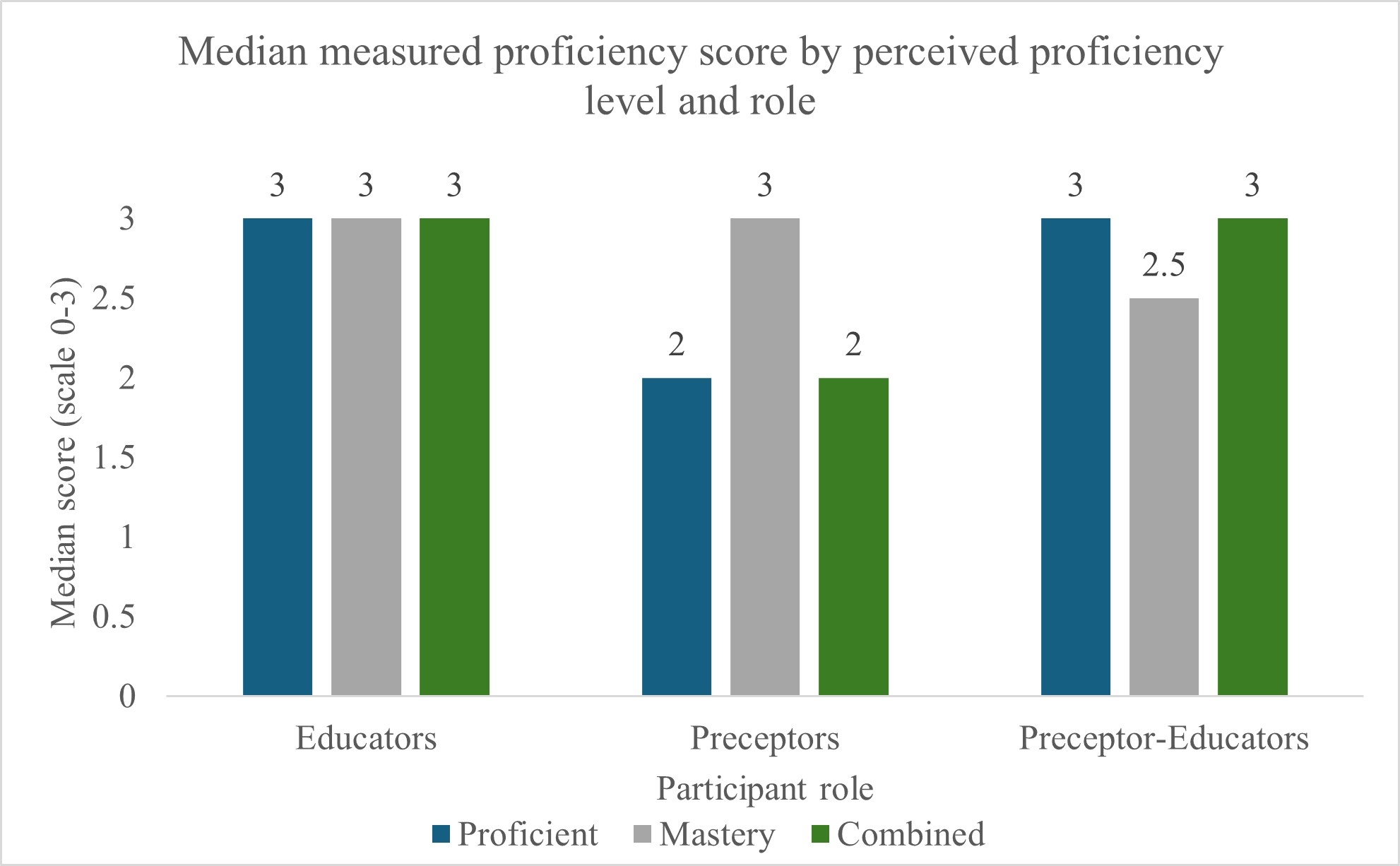

Supplement: SUPPLEMENTARY FIGURE 1 — Median measured proficiency score by perceived proficiency level and role. Median proficiency scores (scale: 0–3) are shown for educators, preceptors, and preceptor–educators across perceived proficiency categories (proficient, mastery, and combined). Educators demonstrated uniformly high median scores, suggesting a ceiling effect, whereas preceptors showed lower and more variable scores. Preceptor–educators exhibited intermediate patterns. [file Image_1.jpg]
