# Supplementary material for: Factors influencing nutrition care process and nutrition care process terminology implementation among United States dietetics educators and preceptors
Source: Front Nutr. 2026 Apr 29;13:1805075. doi: 10.3389/fnut.2026.1805075 (PMC13167533; doi:10.3389/fnut.2026.1805075)
Supplement: Supplementary file 2 [file Table_1.DOCX]

Supplementary Material

# Supplementary Tables

| **Supplementary Table 1. Statistical tests used to examine relationships between independent variables and intention to implement the Nutrition Care Process** | | | | |
| --- | --- | --- | --- | --- |
| Variable Code | Description | Level of Measurement | Statistical Test | Notes |
| D2 | Category | Nominal | Chi-square | Multicategory (Educator, Preceptor, Preceptor-Educator) |
| D3 | Highest level of education | Ordinal | Chi-square for trend / Logistic regression | Ordered categories |
| D4 | Year education completed | Nominal | Chi-square |  |
| D5 | Area of teaching/precepting | Nominal | Chi-square | Select all that apply |
| D6 | State where education completed | Nominal | Chi-square | US states |
| D6R | Region where education completed | Nominal | Chi-square | US states organized into regions (Northeast, Southeast, Midwest, Southwest, and West) |
| D7 | State where practicing | Nominal | Chi-square |  |
| D7R | Region where practicing | Nominal | Chi-square | US states organized into regions (Northeast, Southeast, Midwest, Southwest, and West) |
| D8 | Academy membership | Nominal | Chi-square | Binary (Yes/No) |
| N1 | Heard of NCP | Nominal | Chi-square |  |
| N2 | Perceived NCP competency | Ordinal | Chi-square for trend / Logistic regression | 4-point Likert |
| N3 | Intention to implement NCP | Nominal (Binary) | — | Dependent variable |
| N4 | Negative influences on implementation | Nominal | Chi-square |  |
| N5 | Origin of NCP/T education | Nominal | Chi-square | US vs. non-US, or institution-type |
| N6 | Country NCP/T completed (if outside USA) | Nominal | Chi-square |  |
| P1–P3 | Individual proficiency items | Ratio | t-test / Mann–Whitney U / Logistic regression | Summarized in PS |
| A1–A16 | Individual attitude items | Ratio | t-test / Mann–Whitney U / Logistic regression | Summarized in MAS |
| MAS | Mean attitude score | Ratio | t-test / Logistic regression |  |
| PS | Proficiency score | Ratio | t-test / Logistic regression |  |

| **Supplementary Table 2. Threshold values for failed parametric assumptions.** | | |
| --- | --- | --- |
| Indicator | Test | Acceptable Value |
| Distribution normality | Histogram review | Not applicable |
|  | Skewness | Within +/- 1.0 |
|  | Kurtosis | Within +/- 1.0 |
|  | Kolmogorov-Smirnov test | *p* > 0.05 |
|  | Shapiro-Wilk test | *p* > 0.05 |
| Data independence | Durbin-Watson test | 1.9-2.1 |
| Variance equality | Levene’s test | *p* > 0.05 |
|  | Residual plot | Not applicable |
| Level of measure | Not applicable | Will vary best or aim; dependent variable must be interval or ratio for parametric analysis. |

| **Supplementary Table 3. Free-text responses to variables** | | |
| --- | --- | --- |
| Variable | Response |  |
| N4: Which of the following aspects  have negatively affected the intention  to implement NCP/NCPT into your  practice and teaching? | “VA note template reflects their own expectations”  “Does not impact my daily patient care,  reimbursement or coding”  “Question the relevance and appropriateness”  “Long term care setting is difficult to implement”  “… why add an additional process when we will  not get paid adequately for using it.”  “I don’t have a choice. Because it is essentially  mandated by the Academy.” |  |
| N5: Where did you learn about  NCP/NCPT? | “Employer”  “Implemented into EHR”  “I helped develop it…”  “1 education session with a presentation”  “I don't recall since I've been in practice for 18  years”  “Krause's book”  “Graduate School”  “textbooks, personal research”  “I was in the original people who were trained to  implement it”  “… I had to teach it in a DPD”  “DPD program”  “As an educator having to learn how to teach it to  dietetic students.”  “As a practitioner in a hospital”  “the prior IDNT and current eNCPT”  “I taught myself…”  “Text books” |  |
| Abbreviations: DPD, didactic program in dietetics; EHR, electronic health records; eNCPT,  electronic Nutrition Care Process Terminology; IDNT, International Dietetics Nutrition  Terminology; NCP, Nutrition Care Process; NCPT, Nutrition Care Process Terminology; VA, Veterans Affairs. | |  |

| **Supplementary Table 4. Summary of generative artificial intelligence contributions** | | | |
| --- | --- | --- | --- |
| Project phase | Task | Role | Human verification |
| Data analysis reporting | Review of descriptive and inferential results for clarity and consistency | Identified potential inconsistencies in reported values and denominators | Final data verification, corrections, and statistical decisions made by authors |
| Table development | Drafting and refinement of publication-ready tables (main and supplementary) | Assisted with structuring tables, formatting, and alignment with journal standards | All numerical values verified and approved by authors |
| Manuscript drafting | Language refinement and structural editing of Methods, Results, and Discussion sections | Assisted with wording, flow, and organization of narrative text | Authors retained full control over content, interpretation, and conclusions |
| Manuscript review | Identification of internal inconsistencies and editorial issues | Identification of internal inconsistencies and editorial issues | Authors confirmed and implemented all revisions |
| Statistical reporting | Guidance on appropriate statistical language for abstract and results | Suggested high-level phrasing consistent with reporting standards | Statistical analyses and interpretations conducted by authors |
| Citation formatting | Formatting of reference list in Vancouver style for Frontiers in Nutrition | Reviewed for citation structure, journal abbreviations, and consistency | Authors confirmed accuracy and completeness of references |
| Ethical disclosure | Development of AI-use acknowledgement language | Assisted with drafting disclosure text aligned with journal policy | Authors approved final wording and disclosure placement |
| Generative artificial intelligence was used solely to assist with editorial support, organization, and clarity. No data generation, statistical analysis, or independent interpretation was performed by the model. All final decisions and responsibility for the work rest with the authors. | | | |

# Miscellaneous Supplementary Items

**Investigation Survey**

**Start of Block: Introduction**

Intro Thank you for your interest in this investigation. You are invited to participate in this survey exploring Nutrition Care Process (NCP) and NCP Terminology (NCPT) attitudes among students undergoing supervised practice or a dietetic internship, dietetic educators, and dietetic preceptors in the United States.

**Informed Consent & Frequently Asked Question:**

**Who can participate?** **Please keep in mind that all participants must be adults over the age of 18 years.**

1. All registered dietitian nutritionists (RDN or RD) involved in a United States of America (US)-based Accreditation Council for Nutrition and Dietetics (ACEND) accredited program as an instructor or preceptor
2. Students completing the supervised practice/dietetic internship phase of a US-based

ACEND-accredited program

**Is my participation required?**

No. Your participation is voluntary. You may choose to exit the survey at any time.

**How much time will this require?**

Completion of the survey will require no more 10 minutes for students and 15 minutes for educators.

**Will I be compensated for participation?**

Compensation will not be provided.

**How will my information be collected?**

Responses will be collected and stored in Qualtrics hosted by University of North Florida (UNF), the overseeing organization of this research. Data will be exported onto the passwordprotected, university-secured servers for analysis in Microsoft Excel and IBM SPSS software.

**Do I have to provide any sensitive information?**

No. Personal identification information will not be requested as a part of this investigation.

**Who will see my answers?**

Your responses will be reviewed solely by the team responsible for conducting this research. All answers are anonymous and kept confidential.

**Is there risk involved with participation?**

Participation in this survey is not anticipated to pose any risk, as all data collected as a part of the investigation is anonymized and does not include identification information.

**How will my responses be used?**

Data collected will be used to better understand NCP/T implementation among the groups stated. Results may be presented in academic environments and peer-reviewed publications or presentations. Your participation will help support future investigations that focus on improvement of the NCP/T and development of resources to support its adoption among dietetic practitioners.

**What if I accidently close out my survey?**

If you are unable to resume from where you left off, you can begin a new attempt.

**Is this survey original?**

Though this instrument has not been formally introduced, many of the questions used in this survey were adapted from the International Nutrition Care Process and Terminology Implementation Survey (INIS) developed by Dr. Elin Lövestam and colleagues.(1)

**Are there any supporters to disclose?**

This investigation is funded by the Academy of Nutrition and Dietetics Foundation through the Commission on Dietetic Registration Emerging Researcher Grant.

**Where can I direct my questions?**

Please reach out to Mr. Luc LaBonte via email (n01498228@unf.edu) or phone (252-3209646). To reach the Institutional Review Board (IR) approving this research, please call 904620-2498.

Thanks again for your interest and participation in this research.

Sincerely,

**Reference:**

Lövestam E, Vivanti A, Steiber A, et al. The International Nutrition Care Process and

Terminology Implementation Survey: Towards a Global Evaluation Tool to Assess Individual Practitioner Implementation in Multiple Countries and Languages. J Acad Nutr Diet. 2019;119(2):242-260. doi:10.1016/j.jand.2018.09.004

I1 **Participation in this survey is voluntary. Submission of responses indicates your consent in participation and data usage for research purposes. You are welcome to exit the survey at any time.** o I understand my rights and consent to participate (1) o I do not consent to the methods mentioned (2)

**End of Block: Introduction**

**Start of Block: Demographics**

D1 Which of the following categories best describes your current status? o Registered Dietitian Nutritionist (RD or RDN) (1)

- Graduate Student (not completing supervised practice or dietetic internship) (2) o Graduate Student (completing supervised practice or dietetic internship) (3) o Dietetic Intern (not enrolled in graduate coursework) (4)
- Undergraduate Student (5) o Other (please specify) (6)

__________________________________________________

*Skip To: End of Block If Which of the following categories best describes your current status? = Undergraduate Student*

*Skip To: End of Block If Which of the following categories best describes your current status? = Graduate Student (not completing supervised practice or dietetic internship)*

### Page Break

*Display This Question:*

*If Which of the following categories best describes your current status? = Registered Dietitian Nutritionist (RD or RDN)*

D2 Which of the following categories best describes your role? Select all that apply.

▢ Preceptor (1)

▢ Educator (2) ▢ None of the above (3)

D3 What is your highest level of education you have completed in nutrition or dietetics? o Bachelor's degree (1) o Master's degree (2) o Doctoral degree (3) o Other level of education (please specify) (4)

__________________________________________________

*Display This Question:*

*If Which of the following categories best describes your current status? = Registered Dietitian Nutritionist (RD or RDN)*


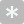


D4 What year did you complete your training to be able to practice as a dietitian?

________________________________________________________________

| *Display This Question:*  *If Which of the following categories best describes your role? Select all that apply. = Preceptor*  *Or Which of the following categories best describes your role? Select all that apply. = Educator* |
| --- |

D5 Which of the following categories do you teach or precept for? Select all that apply.

▢ Patient/client-related (inpatients) (1) ▢ Patient/client-related (outpatients) (2)

▢ Administrative/food service (3)

▢ Administrative/management (13)

▢ Community (e.g. local health promotion, quality routines at nursing homes or schools, work with local food and restaurant suppliers) (4)

| ▢  (12) | Public health (e.g. strategic or policy work at state, provincial or national level) |
| --- | --- |
| ▢ | Consultation and business practice (5) |
| ▢ | Teaching (academic) (9) |
| ▢ | Research (11) |
| ▢ | Other (please specify) (10) |

__________________________________________________

D6 In which state did you complete (or are completing) your dietetic internship/supervised practice?

▼ Alabama (1) ... Other (51)

*Display This Question:*

*If Which of the following categories best describes your current status? = Registered Dietitian Nutritionist (RD or RDN)*

D7 In which state do you practice or teach?

▼ Alabama (1) ... Other (51)

D8 Are you a member of the Academy of Nutrition & Dietetics? o Yes (1)

- No (2)

**End of Block: Demographics**

**Start of Block: NCP Familiarity and Usage**

N1 Have you ever heard of the Nutrition Care Process (NCP)? o Yes (2)

- No (1)

*Skip To: End of Block If Have you ever heard of the Nutrition Care Process (NCP)? = No*

| *Display This Question:*  *If Have you ever heard of the Nutrition Care Process (NCP)? = Yes* |
| --- |

N2 Which category best fits your perceived NCP competency?

- Unfamiliar - lacking the required knowledge and skill to implement (1) o Competent - having the required knowledge and skill to successfully implement (2)
- Proficient - having developed a higher degree of knowledge and skill for aboveaverage implementation (3)
- Mastery - having acquired the highest degree of knowledge and skill for advanced implementation (4)

N3 Do you (or intend to) implement the NCP/T in your practice? o Yes (1)

- No (2)

| *Display This Question:*  *If Do you (or intend to) implement the NCP/T in your practice? = No* |
| --- |

N4 Which of the following aspects have negatively affected the intention to implement NCP/NCPT into your practice and teaching? Select all that apply.

| ▢ | NCP/T does not apply to what I practice, teach, or precept (1) |
| --- | --- |
| ▢ | Lack of confidence with implementation and teaching (2) |
| ▢ | Lack of motivation/ Do not see a reason to change my work approach (7) |
| ▢ | Lack of knowledge (8) |
| ▢ | Lack of time (9) |
| ▢ | Lack of financial resources (10) |
| ▢ | Lack of training and education (11) |
| ▢ | Lack of management support (12) |
| ▢ | Lack of peer support (13) |
| ▢ | Electronic health records unavailable (14) |
| ▢ | Not having access to online tools or books (15) |
| ▢ | Other (please specify) (5) |

__________________________________________________

N5 Where did you learn about NCP/NCPT? Select all that apply.

| ▢ | As a dietetic student/intern in the United States (1) |
| --- | --- |
| ▢ | As dietetic student/intern in another country (2) |
| ▢ | Education sessions with oral presentations (3) |
| ▢ | Education sessions with workshops (4) |
| ▢ | Education sessions with webinars (online seminars) (5) |
| ▢ | Education sessions with simulation (e.g., case study, simulated patients, role |

play) (6)

| ▢ | Departmental meetings with dietetic colleagues (7) |
| --- | --- |
| ▢ | Workplace provided resources (8) |
| ▢ | Was informed by my colleagues who had attended education sessions (9) |
| ▢ | Resources provided by my dietetic association (e.g. website) (10) |
| ▢ | Internet site of the American based Academy of Nutrition and Dietetics (11) |
| ▢ | Internet site of the ICDA (The International Confederation of Dietetic |

Associations) (12)

▢ Other (please specify) (13)

__________________________________________________

*Display This Question:*

*If Where did you learn about NCP/NCPT? Select all that apply. = As dietetic student/intern in another country*


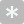


N6 In which country did you learn about NCP/NCPT as a nutrition/dietetics student? ________________________________________________________________

**End of Block: NCP Familiarity and Usage**

**Start of Block: Mini Nutrition Care Process Proficiency Questionnaire**

Assessment Intro **Please answer the following 3 questions to the best of your knowledge.**

P1 By theory, proper implementation of the NCP/T should result in a reduction of which of the following care-related aspects? o Consistency (1) o Autonomy (2) o Patient-centeredness (3) o Ambiguity (4) o I don't know or am unsure (5)

P2 Which of the following is the first concept in the NCP chain? o Evidence (1) o Intervention (2) o Goal (3) o Etiology (4) o I don't know or am unsure (5)

P3 As a monitoring indicator, documented changes in patient/client weight would be best placed in which of the following categories during development of a PES statement? o Problem (1) o Diagnosis (2) o Signs and Symptoms (3) o Etiology (4) o I don't know or am unsure (5)

**End of Block: Mini Nutrition Care Process Proficiency Questionnaire**

**Start of Block: NCP/T Attitudes**

Attitude Intro **Please select your level of agreement for the following statements.**

A1 There are benefits to implementing the NCP (the process). o Strongly disagree (1) o Disagree (2) o Neither agree nor disagree (3) o Agree (4) o Strongly agree (5) o Not applicable (6)

A2 There are benefits to implementing the NCPT (the terminology). o Strongly disagree (1) o Disagree (2) o Neither agree nor disagree (3) o Agree (4) o Strongly agree (5) o Not applicable (6)

A3 The NCP and NCPT allow for clearer documentation. o Strongly disagree (1) o Disagree (2) o Neither agree nor disagree (3) o Agree (4) o Strongly agree (5) o Not applicable (6)

A4 The NCP and NCPT help dietitians to become recognized as more valuable members of interprofessional health-care teams. o Strongly disagree (1) o Disagree (2) o Neither agree nor disagree (3) o Agree (4) o Strongly agree (5) o Not applicable (6)

A5 The NCP provides dietitians with a consistent structure and framework for nutrition care. o Strongly disagree (1) o Disagree (2) o Neither agree nor disagree (3) o Agree (4) o Strongly agree (5) o Not applicable (6)

A6 The NCPT provides dietitians with a common vocabulary to identify nutrition problems. o Strongly disagree (1) o Disagree (2) o Neither agree nor disagree (3) o Agree (4) o Strongly agree (5) o Not applicable (6)

A7 The NCP and NCPT allow for more consistent care when patients/clients are transferred to other care settings. o Strongly disagree (1) o Disagree (2) o Neither agree nor disagree (3) o Agree (4) o Strongly agree (5) o Not applicable (6)

A8 The NCP and NCPT facilitate communication between dietitians. o Strongly disagree (1) o Disagree (2) o Neither agree nor disagree (3) o Agree (4) o Strongly agree (5) o Not applicable (6)

A9 The NCP and NCPT facilitate communication with other health-care professionals. o Strongly disagree (1) o Disagree (2) o Neither agree nor disagree (3) o Agree (4) o Strongly agree (5) o Not applicable (6)

A10 The NCP and NCPT improve patient/client nutrition care. o Strongly disagree (1) o Disagree (2) o Neither agree nor disagree (3) o Agree (4) o Strongly agree (5) o Not applicable (6)

A11 The NCP and NCPT encourage critical thinking among dietitians. o Strongly disagree (1) o Disagree (2) o Neither agree nor disagree (3) o Agree (4) o Strongly agree (5) o Not applicable (6)

A12 The NCP and NCPT facilitate more patient/client involvement in the care process. o Strongly disagree (1) o Disagree (2) o Neither agree nor disagree (3) o Agree (4) o Strongly agree (5) o Not applicable (6)

A13 The NCP and NCPT allow for a holistic perspective on the patients’/client’s situation. o Strongly disagree (1) o Disagree (2) o Neither agree nor disagree (3) o Agree (4) o Strongly agree (5) o Not applicable (6)

A14 The NCP and NCPT help with training of dietetic students/interns during internship/practice placements. o Strongly disagree (1) o Disagree (2) o Neither agree nor disagree (3) o Agree (4) o Strongly agree (5) o Not applicable (6)

A15 The NCP and NCPT support research on patient/client outcomes. o Strongly disagree (1) o Disagree (2) o Neither agree nor disagree (3) o Agree (4) o Strongly agree (5) o Not applicable (6)

A16 The NCP and NCPT support evaluation and development of dietetic practice at the organizational level. o Strongly disagree (1) o Disagree (2) o Neither agree nor disagree (3) o Agree (4) o Strongly agree (5) o Not applicable (6)

**End of Block: NCP/T Attitudes**
